# Pilomatrixoma Recurring as Giant Form

**DOI:** 10.7759/cureus.21308

**Published:** 2022-01-17

**Authors:** Ana Teresa Tavares, Manuel Neiva-Sousa, Carina Semedo, Mariluz Martins, Pedro Gomes

**Affiliations:** 1 Stomathology Department, Hospital de São José, Centro Hospitalar Universitário de Lisboa Central, Lisbon, PRT; 2 Maxilofacial Surgery Department, Hospital de São José, Centro Hospitalar Universitário de Lisboa Central, Lisbon, PRT; 3 Head and Neck Surgery Department, Instituto Português de Oncologia de Lisboa Francisco Gentil, Lisbon, PRT

**Keywords:** head and neck surgery, head and neck tumors, diferential diagnosis, giant pilomatrixoma, skin adnexal tumor, recurrent pilomatrixoma, pilomatrixoma

## Abstract

Pilomatrixoma is a benign skin tumor that originates from the hair matrix. It usually appears in children and young adults and is preferably in the head and neck region. It clinically presents as an asymptomatic firm, solitary subcutaneous mass of less than 3 cm. When located in the preauricular area, it is often misdiagnosed as benign or malignant parotids, skin tumors, or sebaceous cysts. Its treatment of choice is surgery, and recurrence is due to incomplete excision. We present a case of a male referred to our hospital with a diagnosis of recurrent pilomatrixoma in its giant form. The lesion was fully excised with no signs of recurrence and no functional impairment.

## Introduction

Pilomatrixoma is a benign skin tumor that originates from the hair follicle matrix [1-10]. It frequently has a bimodal distribution, with children and young adults having a higher prevalence [1,5,6]. Its frequency is reported as 1:1000 skin biopsies [5] or 1% of all benign skin biopsies [6,9]. It has an unknown etiology, with trauma or insect bites being proposed as causable agents [6]. Clinically, it presents as a solitary, firm, painless subcutaneous slow-growing mass. Since its origin is located superficially, there is usually no adherence to deeper fascias, but its surface can appear reddish/bluish and even ulcerated. Size-wise, it ranges from 0.5 cm to 3 cm [1,2,5,6,8,10], but lesions with a diameter of 34 cm have been described [8]. Therefore, masses larger than 4 cm are classified as "giants" [2,5]. Other classifications include bullous, perforating/ulcerated, anetodermic, lymphangiectatic, pigmented, and familial [2,3,6,9]. Some syndromes are associated with multiple pilomatrixomas, namely Gardner syndrome, Rubinstein-Taybi syndrome, myotonic dystrophy, Kabuki syndrome, trisomy 9, Turner syndrome, and Sotos syndrome [1,3,6,8,9]. Although Turner syndrome and myotonic dystrophy represent the most frequent genetic associations with 42% of cases, no genetic anomalies have yet been established for familial pilomatrixoma [3].

Due to its unspecific presentation, clinical diagnosis is difficult and often misleading. Differential diagnoses include benign and malignant skin tumors, sebaceous cysts, folliculitis, pyogenic granuloma, arteriovenous malformation, lymphoma, and dermatofibroma [1,3-6]. When located in the preauricular area, entities such as benign and malignant parotid neoplasms must be considered because of their clinical resemblance, particularly if the facial nerve is affected due to compression. When presented with ulceration, the malignancy suspicion rises and most hypotheses are cutaneous squamous cell carcinoma, sarcoma, cutaneous metastasis, and malignant parotid tumors [2,5,6,10].

To assess the lesion extension and contour, imaging tests such as computerized tomography (CT), positron emission tomography (PET), magnetic resonance imaging (MRI), and ultrasound may be solicited. Most findings are of a well-defined soft tissue mass with calcifications and no invasion of neighboring tissues. Although these are shared characteristics of other pathologies, in the preauricular area it is of utmost importance to differentiate cutaneous, vascular, and parotid tumors [2,3,5,6,8,10]. PET/CT interpretations should be very cautious, especially regarding large and deep ulcerated lesions, since both high inflammation and a high mitotic rate may increase the glucose intake and therefore reduce the diagnostic accuracy [7]. Fine needle aspiration may help with the diagnosis of pilomatrixoma when the sample contains basaloid and ghost cells with multinucleated giant cells and nucleated squamous cells with calcium deposits [2,3,5,10]. The final diagnosis is given by histopathology when the mass is excised [5,6,8].

Pilomatrixoma does not spontaneously regress, therefore its definite treatment is complete surgical excision [2,5,6,8,10]. Surgical resection depends on the tumor size and nearby structures affected. If preauricular pilomatrixoma’s limits are in close proximity to the parotid gland, superficial parotidectomy may be required. Aesthetics and function can, therefore, be affected despite the presence of a benign entity [2,10]. To reconstruct the surgical defect, several techniques have been reported, such as primary closure, local rotational flap, or skin graft [10].

After removal, the prognosis is very good with recurrency as a very rare event which is owed to incomplete excision [2,5,6,9,10]. Malignancy transformation is extremely rare but has been reported mostly in elderly patients [4-6,9,10].

## Case presentation

**Figure 1:**
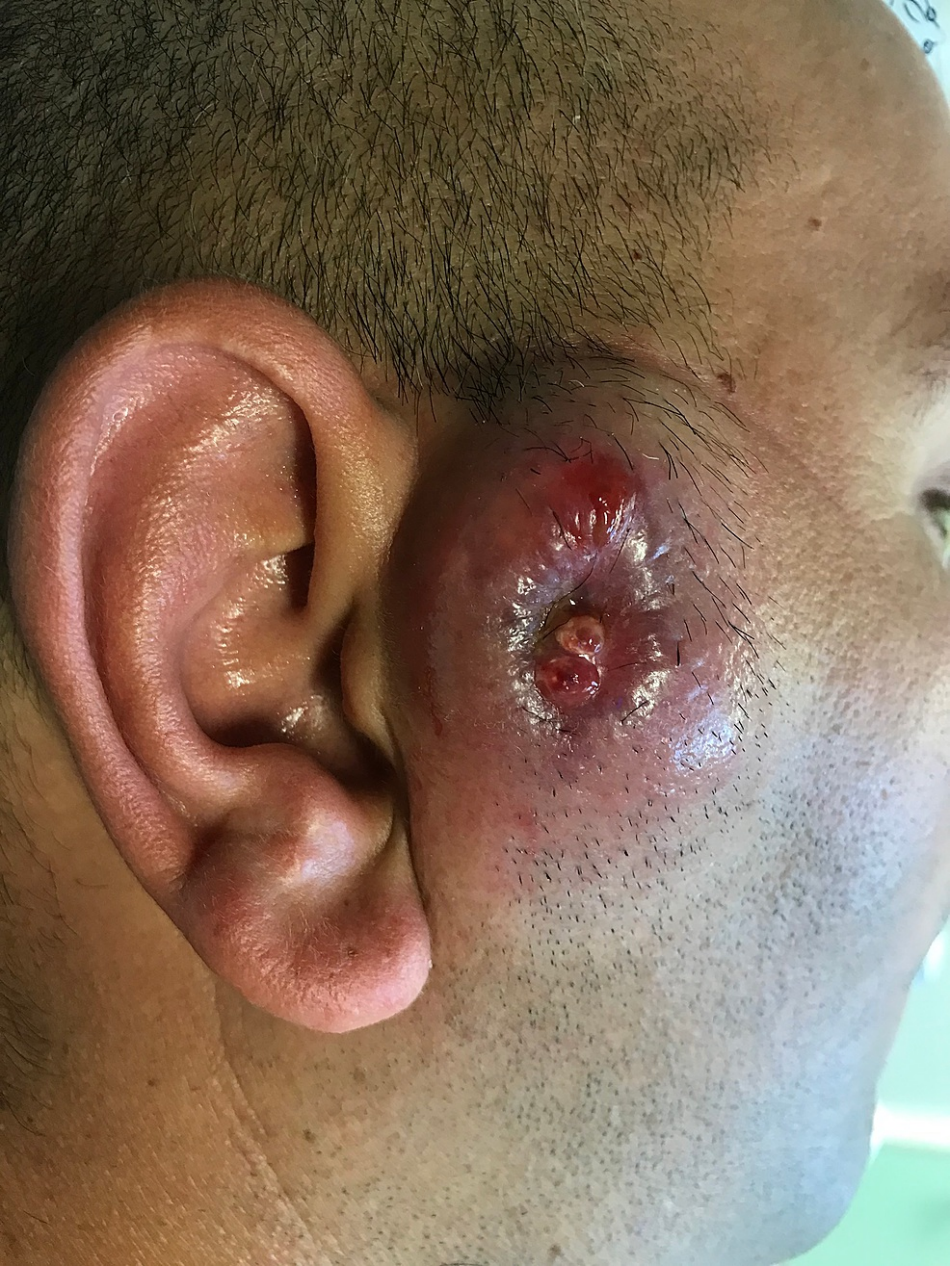
Right preauricular ulcerated nodule at presentation

A healthy 38-year-old male was referred to our hospital with the diagnosis of a recurrent right preauricular lesion that had been excised one month prior, and histopathology revealed "pilomatrixoma." In our observation, a nodule with 4.5 cm and an ulcerated surface was noticed (Figure 1).

**Figure 2:**
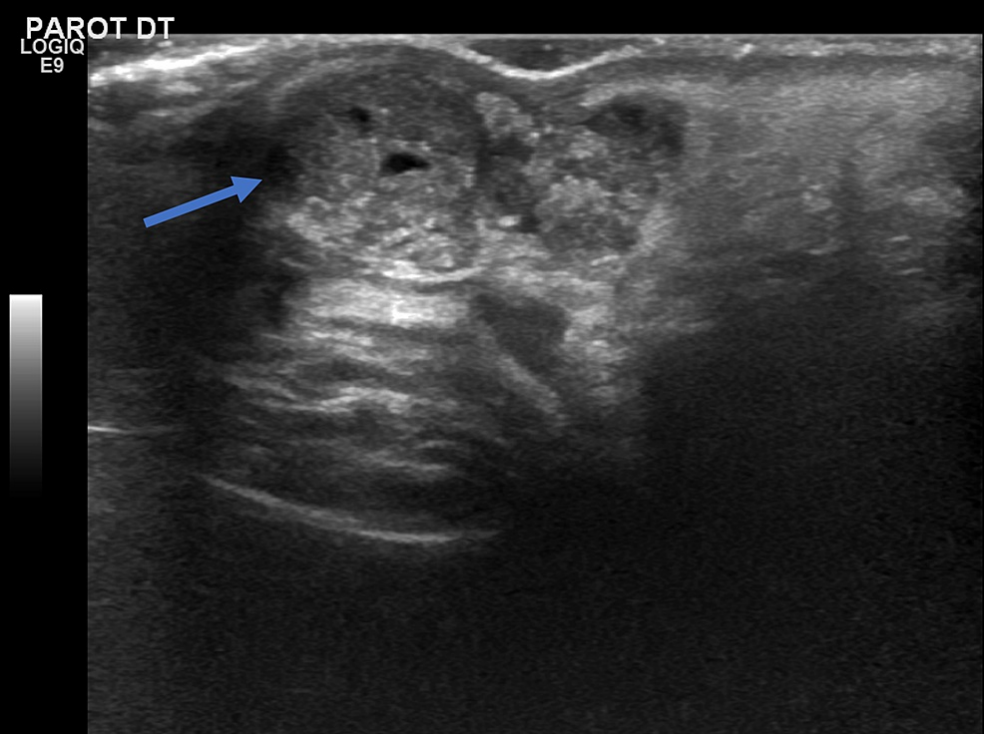
Lesion on ultrasound

It had a firm consistency, adherent to superficial skin, but moved freely in deeper planes. Surrounding the ulcer, there was a reddish halo that was painful on palpation. The facial nerve was unaffected and no lymph nodes were clinically detected. Because of its rapid growth, to help with the definite diagnosis and to ensure the nodule’s relationship with the parotid gland, several exams were requested, namely: histological revision of the previously excised lesion, fine needle aspiration, and parotid ultrasound. A histological revision revealed that the "pilomatrixoma was not completely excised." Fine-needle aspiration reported "epithelial neoplasm with trichilemmal keratinization." The ultrasound revealed a "homogenous oval mass with cutaneous fistula with no relationship to the parotid gland nor facial or cervical lymph nodes detected. The lesion is suspected of being a local recurrence of the previously excised tumor" (Figure 2).

**Figure 3:**
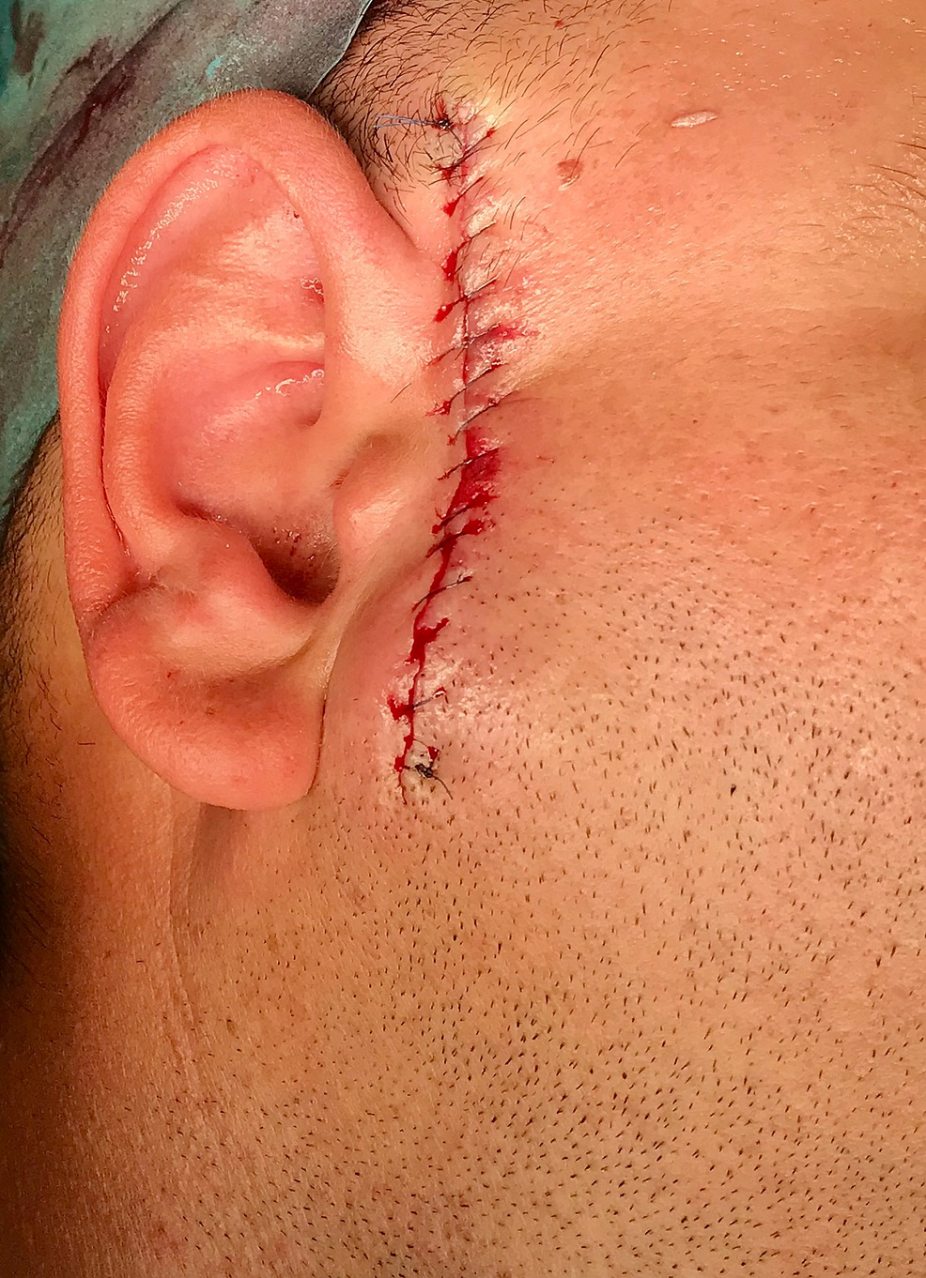
Immediate post-operative suture from primary closure

In conclusion, all the exams coincided with the diagnosis of "recurrent pilomatrixoma." The patient was submitted for complete excision of the lesion, confirmed by histological evaluation of the surgical specimen, with no need for superficial parotidectomy. A macroscopic description of the lesion reported a mass of 4.5 cm x 3.5 cm. The surgical defect was reconstructed with primary closure (Figure 3).

**Figure 4:**
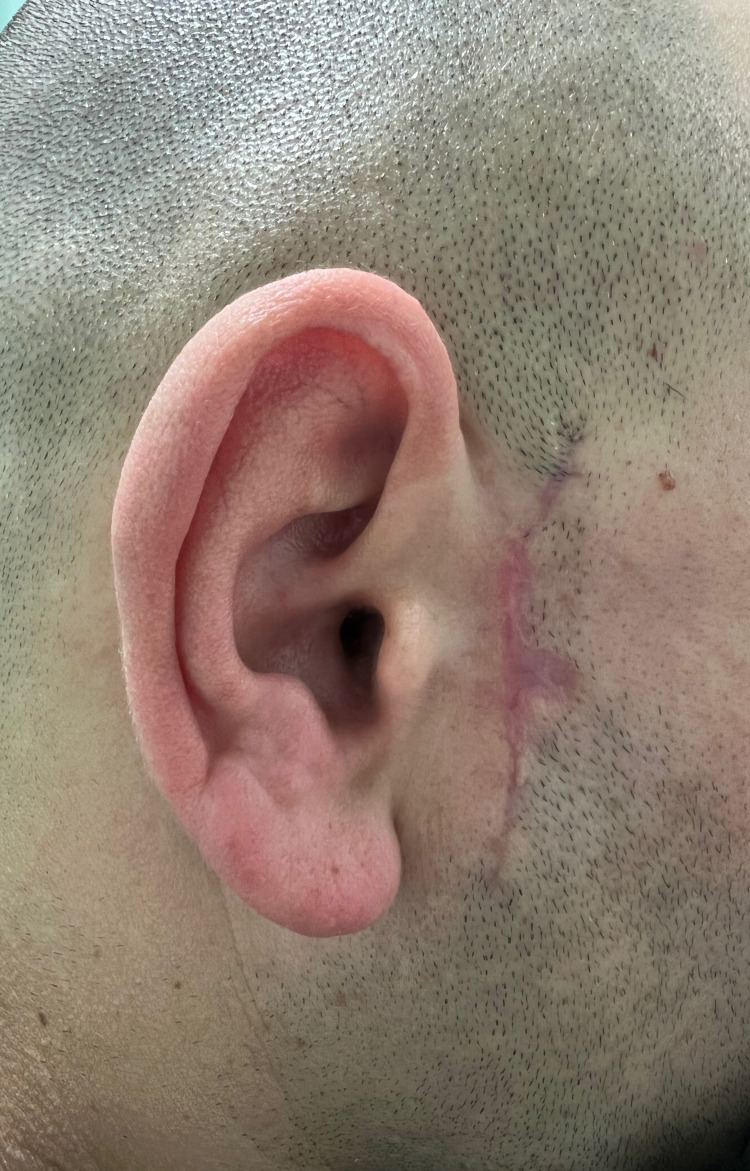
Three months follow-up

On three-month post-op evaluation, the patient remains asymptomatic, with no signs of recurrence, no functional impairment, and a good aesthetic result (Figure 4).

He remains under periodic evaluation with clinical and image control.

## Discussion

Pilomatrixoma is a benign adnexal tumor derived from follicular matrix cells with multiple clinicopathological variants [1-11]. Although averaging 1 cm in size, rare specimens equal to or greater than 4 cm are clustered as giant pilomatrixoma [2,11]. Clinical features are variable, and some may present rapid growth and ulceration. By adding the preauricular location, as reported in this case, pilomatrixoma easily mimics a wide set of other pathologies, invariably including malignant cutaneous or parotid tumors and lymphoma [2,5,11]. As treatment options strikingly differ, a correct diagnosis is of utmost importance and relies on clinical, imagiological, and histopathological findings.

Clinically, the rapid growth following a first excision attempt and the ulcerated reddish skin, depicted in Figure 1, could be interpreted as a sign of malignancy. However, it has been described that these signs can be related to a marked inflammatory process, characterized by a dense perilesional inflammatory infiltrate and reactive lymphadenitis [11].

For imagiological characterization, we selected ultrasound since this method is quick, affordable, does not rely on ionizing radiation, and is suitable for palpable superficial masses. Radiology reported a preauricular homogenous oval mass with a few millimetric cystic areas located in the subcutaneous layer with a cutaneous fistula. According to the literature, common ultrasonographic features of pilomatrixoma comprise a well-defined, oval, hyperechoic nodule-cystic mass located at the subcutaneous level, which is in accordance with our findings [12]. A hypoechoic rim representing a connective tissue capsule surrounding the tumor is also frequently observed [12], although it may be disrupted following incomplete excision and recurrence. The absence of parotid gland involvement was also reported, not only excluding the diagnosis of parotid neoplasm but also eliminating the need for superficial parotidectomy, described as part of the treatment of many pilomatrixomas located in the preauricular area owing to the proximity of the tumor to the parotid gland itself [2].

Of all the methods available, the most reliable for pilomatrixoma diagnosis is histopathological characterization [5]. Previous studies focused on pilomatrixoma histopathology have shown a chronological evolution of the lesion [5,11]. Initially presenting cornified material and ghost cells surrounded by peripheral basaloid cells, the mature pilomatrixoma evolves into a mass of cornified and calcified material containing ghost cells but lacking basaloid cells [11]. In this case report, histopathological characterization was based on fine-needle aspiration and described as an epithelial neoplasm with trichilemmal keratinization, compatible with pilomatrixoma. The absence of calcified material was expected in a recent recurring lesion. Pilomatrixoma treatment involves complete surgical resection of the tumor [2,4,6]. Recurrence is rare and is associated with incomplete excision [2,5,6,9,10]. In this case report, revision of blades from the first excision confirmed the absence of tumor-free margins, justifying the relapse. Finally, surgical treatment of some giant pilomatrixomas requires reconstruction with local flaps [2]. In this case report, however, direct closure following resection was possible, with an acceptable aesthetic outcome (Figure 3 and 4).

## Conclusions

Pilomatrixoma is a benign entity easily mistaken for malignant tumors. Correct identification of the lesion relies on clinical, imagiological, and histopathological findings and allows a much less aggressive surgical approach. Recurrence is rare, but possible if excision is incomplete. Here, we reported a case of a pilomatrixoma of the preauricular area recurring as a giant form.
